# Describing implementation outcomes for a virtual community of practice: The ECHO Ontario Mental Health experience

**DOI:** 10.1186/s12961-022-00818-1

**Published:** 2022-02-08

**Authors:** Eva Serhal, Cheryl Pereira, Rosaria Armata, Jenny Hardy, Sanjeev Sockalingam, Allison Crawford

**Affiliations:** 1 Virtual Care, Outreach, and ECHO Department, Centre for Addiction and Mental Health, Toronto, ON Canada; 2 Department of Education, Centre for Addiction and Mental Health, Toronto, ON Canada; 3 Department of Psychiatry, Faculty of Medicine, University of Toronto, Toronto, ON Canada

**Keywords:** Tele-education, Mental health, Capacity-building, Project ECHO, Implementation science, Implementation outcomes

## Abstract

**Background:**

Project ECHO is a virtual education model aimed at building capacity among healthcare providers to support optimal management for a range of health conditions. The expansion of the ECHO model, further amplified by the pandemic, has demonstrated an increased need to evaluate implementation success to ensure that interventions are implemented as planned. This study describes how Proctor et al.’s implementation outcomes (acceptability, adoption, appropriateness, costs, feasibility, fidelity, penetration, and sustainability) were adapted and used to assess the implementation of ECHO Ontario Mental Health (ECHO-ONMH), a mental health-focused capacity-building programme.

**Methods:**

Using Proctor et al.’s implementation outcomes, the authors developed an implementation outcomes framework for ECHO-ONMH more generally. Using this, outcome measures and success thresholds were identified for each outcome for the ECHO-ONMH context, and then applied to evaluate the implementation of ECHO-ONMH using data from the first 4 years of the programme.

**Results:**

An ECHO-ONMH implementation outcomes framework was developed using Proctor’s implementation outcomes. ECHO-ONMH adapted implementation outcomes suggest that ECHO-ONMH was implemented successfully in all domains except for penetration, which only had participation from 13/14 regions. Acceptability, appropriateness and adoption success thresholds were surpassed for all 4 years, showing strong signs of sustainability. The programme was deemed feasible all 4 years and was found to be more cost-effective. ECHO-ONMH also showed high rates of fidelity to the ECHO model, and high rates of penetration.

**Conclusions:**

This is the first study to use Proctor et al.’s implementation outcomes to describe implementation success for a virtual capacity-building model. The proposed ECHO implementation outcomes framework provides a base for similar interventions to evaluate implementation success, which is an important precursor to understanding learning, service or health outcomes related to the model. Additionally, these findings can act as a benchmark for other international ECHOs and educational programmes.

## Background

Project Extension for Community Healthcare Outcomes (ECHO^®^) is a mature and validated model of virtual education and capacity-building that has shown excellent spread and scale globally. It was developed at the University of New Mexico to address regional variances in access to specialty care, in particular improving access for people living in rural and remote underserved regions [1, 2]. The ECHO model uses a “hub-and-spoke” approach to share best practices from diverse clinical experts in a specific field (the hub) with community-based primary care providers (PCPs) that are regionally dispersed (the spokes). Through regularly scheduled multipoint videoconferencing sessions that combine case-based learning with mini-didactics focused on evidence-based best practices in care, Project ECHO provides an interactive space for over 449,000 spokes in diverse global locations [1–3].

The international spread of the ECHO model was sparked by an influential study published by Project ECHO founder Dr Sanjeev Arora and colleagues in 2011, suggesting that in many cases, PCPs supported by tele-education and tele-mentoring via ECHO were able to provide care that led to equivalent or better outcomes when compared to specialist care for patients with hepatitis C [2]. Since then, the ECHO model has expanded significantly, with over 49 countries delivering 955 ECHO projects across a range of areas within healthcare (including behavioural/mental health, chronic pain, HIV, geriatrics and cancer care) and beyond [2, 4–6]. Numerous studies have shown that ECHO expands PCP knowledge of best practices, increases self-efficacy and behaviour, and improves patient outcomes as well as cost-effectiveness [1, 7–10] Additionally, several qualitative studies have suggested that ECHO programmes can serve to reduce providers’ feelings of isolation or help create a community of practice [11, 12].

More recently, the model has seen rapid proliferation in a variety of contexts to disseminate rapidly evolving information related to public health, while supporting healthcare providers and facilitating a sense of community at a distance. Currently, 260 ECHO projects within 33 countries are supporting efforts to disseminate knowledge on COVID-19 [13, 14].

The ECHO community, led by the ECHO Institute, has continued to support sustainability through system and policy change at state and federal levels, foundation grants, and integration within the healthcare system [15]. As the model continues to expand and replicate, assessing implementation outcomes is essential; implementation outcomes are important mediating outcomes that help to establish whether expected patient and provider outcomes can truly be attributed to the intervention or innovation [16]. While the majority of ECHO projects evaluate items such as satisfaction or participation, many do not adopt a full implementation science lens to assess overall project implementation [7, 8, 10, 17].

ECHO is founded on four key principles that each programme must comply with to ensure fidelity to the model: (1) use technology to leverage scarce resources; (2) share best practices to reduce disparity; (3) use case-based learning to master complexity; and (4) monitor outcomes. Finally, when applied together, these principles result in an “all teach and all learn” environment in which all members of the community (hub and spokes) participate in both the sharing and gaining of knowledge in ECHO sessions [2]. The ECHO Institute in New Mexico takes numerous steps in order to help support fidelity to this model, such as the requirement for signed agreements outlining use terms for calling a project an “ECHO”; immersion training, which is a 3-day training session that includes training on the model and support with programme planning; and Meta-ECHO virtual calls and a Meta-ECHO conference that bring together ECHOs from all over the world to share information, ideas and quality improvement initiatives. The growth of ECHO and the ability for rapid implementation is facilitated by a rigorous replication model, which requires fidelity to the four key principles instead of processes, while enabling sufficient flexibility and adaptability of the model. Implementing new projects such as ECHO can be complex; therefore, assessing organizational readiness and evaluating implementation success can ensure that projects are implemented as planned, reducing wasted time, cost and effort [18–21].

ECHO Ontario Mental Health (ECHO-ONMH) at the Centre for Addiction and Mental Health (CAMH) is a large ECHO programme that is funded by Ontario’s Ministry of Health. It has been operational since 2015, and currently runs 11 different sub-ECHO projects, including a recently implemented ECHO Coping with COVID project, aimed at supporting healthcare providers throughout Canada as they manage the COVID-19 pandemic [22].

This study utilized Proctor et al.’s implementation outcomes paired with expertise of the ECHO model [16] to create a framework for measuring implementation of ECHO-ONMH and to assess the implementation success of ECHO-ONMH during its first 4 years. Proctor et al.’s implementation outcomes model consolidates approaches from diverse implementation science frameworks to create a common taxonomy for measuring the implementation of interventions [16]. The framework proposes eight core implementation outcomes (acceptability, adoption, appropriateness, costs, feasibility, fidelity, penetration and sustainability). These outcomes have been widely used to evaluate implementation success of e-health technologies including those focused on mental health [23]. Research using other implementation science frameworks such as the Consolidated Framework for Implementation Research has been conducted to evaluate key implementation facilitators and barriers for ECHO [24]; however, the lack of approaches for measuring the implementation success of an ECHO programme or similar virtual training models is a notable gap in the literature. The following study describes how Proctor et al.’s eight implementation outcomes can be adapted to assess the implementation of ECHO projects or other similar virtual educational models [16].

## Methods

### Study design

We employed a descriptive study design leveraging Proctor et al.’s implementation outcomes framework to explore the implementation of ECHO-ONMH [16, 25, 26]. Definitions and examples of measures articulated in the framework were utilized to formulate a set of aligned implementation outcome measures for ECHO-ONMH, and then used to identify measures and thresholds to assess the implementation of ECHO-ONMH from 2015 to 2019. Measures for each outcome were identified by reviewing retrospective ECHO-ONMH programmatic measures and accompanying data (routinely collected as part of programme metrics for quality improvement and reporting requirements). Where no measures for a particular outcome existed, such as cost, the team discussed the most meaningful way to measure the outcome within this study, including the use of evidence-based assumptions. All evaluation measures and data sets used in this study are in accordance with the CAMH Quality Project Ethics Review.

### Setting

ECHO-ONMH is a tele-education programme that was adapted for the mental health context and implemented in 2015 at CAMH to support PCPs providing general mental healthcare across Ontario, Canada, especially those located in areas where access to specialists might be scarce. It is fully funded by Ontario’s government, and as part of the funding agreement, the ECHO-ONMH programme is required to meet certain programme deliverables, as established by the funder. An interdisciplinary specialist (hub) team at CAMH connects with numerous PCPs (spokes) from across the province of Ontario for 2 hours on a weekly basis to form a virtual community of practice, where all can share best practices. Each session begins with a brief didactic lecture focused on varied mental health conditions including mood disorders, anxiety disorders, psychotic disorders, substance use disorders, and trauma- and stressor-related disorders. The remainder of the ECHO session is focused on spoke and hub discussion of complex anonymized patient cases from participating spoke practices. At the end of ECHO sessions, participants have gained new information about best practices in mental healthcare, and have been able to apply their learning to a real-life practice-based case scenario. The ECHO-ONMH hub consists of interprofessional subject matter experts within the field of mental health and addiction, which includes general psychiatrists, a child and youth psychiatrist, an addiction specialist, a family physician, a social worker and a librarian. Participants (spokes) in the ECHO-ONMH programme are interprofessional healthcare providers from diverse settings across Ontario providing mental healthcare. The programme is supported by an operations team that coordinates the ongoing logistics of the programme. ECHO-ONMH delivers between 32 and 36 2-hour sessions on a weekly basis per annual cycle. This study analysed the implementation outcomes for the first four cycles of the programme held between 2015 and 2019.

In order to participate in ECHO-ONMH, PCPs complete an online registration form and sign a statement of collaboration (SoC). The registration form captures key professional demographic and participant information, and the SoC establishes guidelines for participation. Of note, the SoC states that participants are required to attend as many sessions as they can, barring known or unavoidable absences such as sick or vacation days. Participants commit to attending 70% (cycles 1 and 2) and 60% (cycles 3 and 4, lowered based on experiences from cycles 1 and 2) of total sessions in order to register for the programme.

### Outcome measures and data sources

**Fig. 1:**
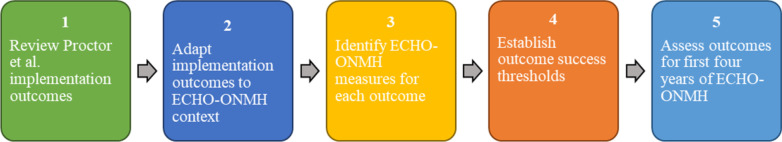
Process for measuring implementation of ECHO-ONMH

Two of the authors (ES and JH) first conducted a detailed review of the proposed implementation outcomes, including the theoretical basis of each outcome and the original “taxonomy of implementation outcomes”, which provides a list of potential measurement options and recommendations [16]. Using the taxonomy of implementation outcomes, the two authors discussed ways in which each outcome could be adapted to measure ECHO-ONMH. The ECHO-ONMH research and operations team (CP and RA) subsequently generated a list of available measures and sources that were routinely used in ECHO-ONMH programme evaluation and quality improvement data collection and analyses to see whether any of them fit the criteria for measurement of the implementation outcomes. Using identified ECHO-ONMH data sources, the team developed a framework with measures and thresholds for implementation success for each outcome. Measures for each outcome were discussed by all authors to ensure fit with the implementation outcome definition and measurement suggestion in Proctor et al.’s implementation outcomes framework. The measures were only included if full consensus was reached around alignment [16]. Thresholds for success were determined using funding and programme requirements established by the programme funder, expectations set by the hub team for success, expectations for participation by the spoke sites (including those set out in the SoC), expectations for fidelity and replication set out in partnership agreements with the ECHO Institute, and statistics released by the ECHO Institute representing benchmarks at an international level. In order to ensure that no bias was introduced, the authors determined which measures to use and thresholds for implementation outcome success prior to looking at the data sets. Once initial measures and thresholds were identified, they were shared with ECHO subject matter experts, including the ECHO Institute in New Mexico, members of the ECHO Ontario Superhub, and ECHO-ONMH co-chairs for feedback and alignment around definitions and thresholds. Feedback was incorporated through an iterative process, and the expert group validated final versions of the measures. A summary of the process can be seen below in Fig. 1.

Measures and data from the following sources were used to evaluate each outcome: ECHO-ONMH registration forms; weekly anonymized ECHO session satisfaction surveys; weekly session attendance logs; and video recordings from ECHO-ONMH sessions. Frequencies and proportions for demographic categorical variables (i.e. professions, primary practice settings, geographical regions and number of registrants) were calculated for each cycle. Weekly session attendance logs were maintained in ECHO-ONMH to track participation rates and trends for each cycle.^1^ As a routine programme evaluation activity for ECHO-ONMH, participants are sent links for online-anonymized weekly satisfaction surveys after every ECHO session. Surveys include statements on satisfaction and learning needs, which are responded to using a five-point Likert scale (1 = strongly disagree and 5 = strongly agree). After the end of each cycle, mean scores and standard deviations were tabulated for all domains in the survey. Video recordings of ECHO-ONMH sessions held during cycles 1 to 4 were randomly selected, viewed and scored by a research analyst using the five fidelity factors defined by the ECHO Institute. The research analyst was trained on and had strong familiarity with the ECHO model (i.e. had participated in ECHO orientation and immersion training). Any concerns around scoring were brought to the primary investigator for further discussion and resolution.

Descriptions of the eight implementation outcomes as well as implementation measures and success thresholds for ECHO-ONMH are described below and summarized in Table 1.

*Acceptability* considers how agreeable, palatable or satisfactory the innovation is to its stakeholders [16]. Within the context of ECHO-ONMH, acceptability looks at how satisfied PCPs are with the programme. The authors reviewed existing ECHO-ONMH evaluation measures that assessed participants’ satisfaction with the programme, and identified the measure “overall, I was satisfied with the session” on a five-point Likert scale, which is a single item within the weekly session satisfaction surveys. The mean score for this statement across each cycle was examined. An ECHO-ONMH cycle was considered acceptable if it held a mean score of ≥4/5 (i.e. the “acceptability threshold”), which indicated that on average, participants were satisfied or highly satisfied with the cycle.

*Adoption* examines the uptake of a practice or innovation by an individual or organization, including both intent to try and action itself [16]. Within the context of ECHO-ONMH, adoption is the extent to which PCPs utilized the programme. The authors examined trends in programme registration and participant attendance within an ECHO cycle to help understand intent to try and the action of joining. Intent to try was measured by the number of PCPs who registered for the programme each cycle. Action was measured by the number of participants who attended one or more sessions per cycle (i.e. those on the “roster”), as well as the average number of participants per session in a cycle. Thresholds for adoption were informed by the ECHO-ONMH programme funding agreement, which states that ECHO-ONMH must maintain a roster of 20 participants in a cycle, and have a minimum of 6 participants per session. The threshold for intent to try was developed based on anticipated attrition rates in ECHO-ONMH, which can reach up to 25% from registration to actual participation. As a result, we established a threshold of a minimum of 25 registrants for each cycle for intent to try (25% higher than a roster of the required 20 participants). The threshold for action used the direct requirements outlined in the ECHO-ONMH programme funding agreement, where a minimum of 20 participants (i.e. individuals who attended ≥ 1 sessions) must be maintained in a cycle, with a minimum of six participants per session. An ECHO-ONMH cycle was successful in the adoption outcome if it met both intent to try and action thresholds.

*Appropriateness* is the perceived fit, compatibility and relevance of an innovation to an individual’s or organization’s problem, challenge and/or setting [16]. Within the context of ECHO-ONMH, appropriateness is the fit of the programme in addressing the learning needs of participants. We looked for a measure that examined participants’ feelings around perceived learning gained from their participation in ECHO, and identified the measure “this session has addressed my learning needs” on a five-point Likert scale, which is a single item within the weekly session satisfaction surveys. The mean score for this statement across each cycle was examined. An ECHO-ONMH cycle was considered appropriate if it held a mean score of ≥4/5 (i.e. the “appropriateness threshold”), which indicated that, on average, ECHO sessions addressed participants’ learning needs.

*Cost* is the pricing of the intervention and its implementation. Is it less expensive than other options [16]? A full economic analysis or cost modelling would likely warrant and require a study all its own, and includes very robust analysis. In order to assess this outcome within the context of the framework and study, the authors developed a basic cost comparison (in Canadian Dollars (CAD)) using assumptions for how much it would cost the healthcare payer and participant to participate in ECHO-ONMH sessions (and receive accredited continuing medical education [CME] hours) compared to the cost of participating in an in-person CME event, such as a conference event in Toronto, Ontario, with comparable CME hours. Assumptions are listed in the notes section of Table 2. Cost comparison for the ECHO model compared to an in-person conference in Toronto was evaluated on (1) per-person cost to participate (estimated at 72 CME hours), (2) total cost per CME hour and (3) total cost for all individuals who participated in ECHO-ONMH during the study period (using total number of participants across four ECHO-ONMH cycles). The threshold for success with respect to cost was if costs to participate in ECHO-ONMH for all three cost comparisons was less than costs to participate in an in-person conference.

*Feasibility* considers whether an innovation is practical for a provider and/or organization, shaping whether it can be implemented [16]. Within the context of ECHO-ONMH, feasibility looks at whether the programme is practical for PCPs to participate in. We examined trends in participant attendance within an ECHO cycle to assess how feasible it was for PCPs to participate in ECHO-ONMH. We assessed this using the average number of sessions participants attended per cycle. An ECHO-ONMH cycle was considered feasible if the average number of sessions participants attended per cycle was equal to or greater than the global average from the ECHO Institute (average of 6 sessions) between the study period of September 2015 and June 2019 [4]. Previously tabulated averages for each cycle were reviewed and compared to the global average.

The authors also conducted a secondary exploratory feasibility analysis looking at the proportion of participants that were able to meet the attendance requirement of ≥ 60% (as per our most recent attendance requirement outlined in the SoC) of sessions per cycle, and what their professions, practice types and regional areas of practice were. No threshold was established for this exploratory analysis.

*Fidelity* considers the actual implementation compared to that which was prescribed by a particular protocol or model to determine adherence, quality and integrity [16]. Within the context of ECHO-ONMH, fidelity looks at whether the programme was delivered as intended in the ECHO model (as set out by the ECHO Institute). This was evaluated by measuring each cycle’s adherence to the four key principles of the ECHO model, as well as the presence of the “all teach, all learn” environment. Criteria for evaluation included the following:

1. Best practices—Were education and best practices shared through a didactic presentation?
2. Case-based learning—Was there a case presented by a community partner to support the case-based learning component?
3. Technology—Was videoconferencing technology leveraged for sessions?
4. Assess outcomes—Were session outcomes measured (i.e. is there an evaluation after each session)?
5. All teach/all learn—Did both the hub and spokes share/interact with one another?

Five video-recorded sessions from each cycle were randomly selected and evaluated for adherence to the principles and presence of the all teach/all learn environment using a table with a binary scale (1 = yes, 0 = no). An ECHO-ONMH cycle achieved fidelity if all principles (i.e. 5/5) were met for all randomly selected videorecorded sessions for each cycle.

*Penetration *is the integration or spread of a particular service, practice or innovation to its potential settings and subsystems [16]. Within the context of ECHO-ONMH, penetration is the extent to which the programme is reaching its target settings. It is essential that the ECHO-ONMH reaches a vast number of geographical regions to address the problem of inequitable access to mental health and addiction care in the province. As such, we looked at the measure of regional reach using the province’s 14 local health integration networks (LHINs). LHINs are artificial regions within the province, and are established to support the funding, planning and delivery of care. The proportion of LHINs reached over time was analysed to understand penetration. Penetration was achieved when 100% of the LHINs (i.e. 14 out of 14 regions) had spoke sites registered in ECHO-ONMH.

*Sustainability* is whether the innovation is maintained or established as an ongoing, institutionalized offering. Within the context of ECHO-ONMH, sustainability was evaluated by assessing the number of years that minimum adoption was sustained (each cycle has a minimum of 25 PCPs registered, a minimum roster of 20 participants and a minimum average of six participants per session). ECHO-ONMH was considered sustainable if it met minimum adoption over the years assessed. Funding is another important factor to consider for sustainability; this programme would continue to sustain ongoing annual funding if it met the thresholds described above.

## Results

As described in the methods section, we first reviewed Proctor et al.’s implementation outcomes, and then adapted each outcome for the ECHO-ONMH context. Following this, we identified measures within ECHO-ONMH to assess the ECHO-adapted implementation outcomes, and finally established a threshold for success to determine whether we succeeded within the context of each particular implementation outcome. Table 1 below maps out these items, and the paragraphs below summarize the results of our first 4 years of the ECHO-ONMH programme within the context of the adapted implementation evaluation framework.

**Table 1:** Summary of implementation measures and success thresholds for ECHO-ONMH

Implementation outcome definitions [16]	Implementation outcome adapted for ECHO-ONMH	Description of implementation measures for ECHO-ONMH	Description of proposed implementation success thresholds for ECHO-ONMH	Summary of implementation success for ECHO-ONMH based on threshold
Acceptability: how agreeable, palatable or satisfactory the innovation is to its stakeholders	Participants’ satisfaction with ECHO	Mean score for full cycle of weekly session satisfaction survey statement “Overall, I am satisfied with the session” on a 5-point Likert scale	Achieves a mean score of ≥ 4/5 each cycle, indicating ECHO-ONMH is acceptable (satisfactory) by participants’ self-report	All four cycles of ECHO-ONMH (100%) met the threshold, demonstrating high levels of acceptability among participants
Adoption: uptake of a practice or innovation by an individual or organization, including both intent to try and action itself	Utilization of ECHO by participants (intent to adopt and act of adopting)	Intent to adopt: number of PCPs that registered for ECHO-ONMH each cycle Act of adopting: number of participants who attend ≥ 1 ECHO-ONMH sessions within a given cycle, and average number of participants in attendance per session (i.e. action per session) within a given cycle	Achieves ≥ 25 PCPs registered (intent to try), as well as achieves ≥ 20 participants attending ≥ 1 session(s) and an average of ≥ 6 participants per session (action) each cycle	All four ECHO-ONMH cycles (100%) met both thresholds, thereby considered successful in the adoption outcome
Appropriateness: perceived fit, compatibility and relevance of an innovation to an individual’s or organization’s problem, challenge and/or setting	Relevance of ECHO curriculum/whether sessions meet participant learning needs	Mean score for weekly session satisfaction survey statement “this session addressed my learning need” on a 5-point Likert scale	Achieves a mean score of ≥ 4/5 each cycle, indicating ECHO-ONMH is appropriate (meeting learning needs), as identified by participants’ self-report	All four cycles of ECHO-ONMH (100%) met the threshold and are considered successful in the appropriateness outcome
Cost: pricing of the intervention and its implementation. Is it less expensive than other options?	Estimate of cost to participate in ECHO compared to in-person CME conference	Cost comparison (in CAD) for the ECHO model compared to an in-person conference in Toronto, comparing: Per-person cost to participate (estimated at 72 CME hours), total cost per CME hour and total cost for all individuals (using total number of participants across 4 ECHO-ONMH cycles)	Costs to participate in ECHO-ONMH for all three cost comparisons is ≤ costs to participate in an in-person conference	The cost savings for an individual to participate in a cycle of ECHO-ONMH is about $25 per CME hour, $1833 per annual cycle, and $747 864 by model/programme per year. No success threshold, but this does constitute a significant cost savings for both the individual and the public healthcare funder
Feasibility: considers whether an innovation is practical for a provider and/or organization, shaping whether it can be implemented	How practical is ECHO for participants to participate	Average number of sessions participants attend within a given cycle Further exploratory analysis will look at attendance rates by profession, practice types and practice location	The average number of sessions participants attend in each cycle is ≥ the global average from ECHO Institute for each cycle (6 sessions) [4] Threshold is not established for stratified attendance, as this analysis is exploratory in nature	All four ECHO-ONMH cycles (100%) met the threshold and are considered successful for the feasibility outcome
Fidelity: actual implementation compared to that which was prescribed by a particular protocol or model to determine adherence, quality and integrity	Fidelity to the ECHO model as identified by the ECHO Institute	Adherence to four global ECHO principles and the presence of an “all teach/all learn” environment within a random sample of five videorecorded sessions per ECHO-ONMH cycle: Best Practices Case-Based Learning Technology Assess Outcome All Teach/All Learn	100% of the fidelity criteria (adherence to principles and presence of all teach/all learn environment) are met across video samples selected for each cycle	All four ECHO-ONMH cycles (100%) met the threshold, having exceptionally high rates of fidelity to the Project ECHO model (adhered to 100% of criteria)
Penetration: integration or spread of a particular service, practice or innovation to its potential settings and subsystems	Reach across all targeted regions in Ontario	Proportion of Ontario’s 14 regional area LHINs reached (as represented by participants) in each cycle. LHINs are artificial regions that support the funding, planning and delivery of care, as the basis for the regional areas	Achieves registration from 100% of the LHINs (i.e. 14 out of 14 regions) in each cycle	None of the four cycles of ECHO-ONMH (0%) met the threshold and achieved successful penetration
Sustainability: innovation is maintained or established as an ongoing, institutionalized offering	Sustained adoption across cycles (required in order to meet funding agreements and sustain funding)	Number of years minimum adoption was sustained (≥ 25 PCPs registered, a roster of ≥ 20 participants, and a minimum average of 6 participants per session)	Meets the adoption threshold for each of the four cycles (2015–2019)	All four cycles of ECHO-ONMH (100%) met the threshold. ECHO-ONMH can be seen as a sustainable programme

The first four ECHO-ONMH cycles included in this study consisted of 32, 33, 33 and 36 2-hour weekly sessions for cycles 1, 2, 3 and 4, respectively. Cycles 1, 2, 3 and 4 had 146, 113, 58 and 91 participants (individuals who attended ≥ 1 sessions). Descriptive statistics for weekly session surveys were tabulated based on 309 (30.1% average response rate), 366 (40.6%), 331 (43.9%) and 598 (40.4%) responses received for cycles 1, 2, 3 and 4, respectively. The five randomly selected coded videos for each cycle made up 15.6%, 15.1%, 15.1% and 13.9% of the video sample for cycles 1, 2, 3 and 4, respectively.

### Acceptability

Acceptability (or overall satisfaction) surpassed the threshold for success of 4/5 across all four cycles of the programme. In cycle 1 (2015–2016), the mean satisfaction rating was 4.28/5 (SD = 0.31, *n* = 309 survey responses), in cycle 2 (2016–2017) the mean satisfaction rate was 4.45/5 (SD = 0.18, *n* = 366), in cycle 3 (2017–2018) the mean satisfaction rate was 4.31/5 (SD = 0.21, *n* = 331) and in cycle 4 (2018–2019) the mean satisfaction rate was 4.21/5 (SD = 0.19, *n* = 598). Based on our established success threshold for acceptability (mean rating of ≥ 4/5), all four cycles of ECHO-ONMH demonstrated high levels of acceptability (satisfaction) among participants.

### Adoption

In cycle 1, 196 people registered, 146 participants attended ≥ 1 session(s), and the average attendance per session was 33.31 (SD = 6.64) participants. In cycle 2, 141 people registered, 113 participants attended > 1 session(s), and the average attendance per session was 27.85 (SD = 8.10) participants. In cycle 3, 69 people registered, 58 participants attended > 1 session(s), and the average attendance per session was 22.03 (SD = 5.02) participants. In cycle 4, 100 people registered, 91 participants attended > 1 session(s), and the average attendance per session was 39.89 (SD = 12.55) participants. Based on the established thresholds (each cycle to have a minimum of 25 PCPs registered, a roster of minimum 20 participants, and a minimum average of 6 participants per session), all four ECHO-ONMH cycles are considered successful in the adoption outcome.

### Appropriateness

Mean scores for appropriateness (participants’ perceptions that their learning needs are being met in ECHO) surpassed the threshold for success across all four cycles of the programme. In cycle 1, the mean participant rating for the question “this session has addressed my learning needs” was 4.15/5 (SD = 0.28, *n* = 309 survey responses); in cycle 2, the mean participant rating was 4.28/5 (SD = 0.24, *n* = 366); in cycle 3, the mean participant rating was 4.17/5 (SD = 0.22, *n* = 331); and in cycle 4, the mean participant rating was 4.12/5 (SD = 0.23, *n* = 598). Based on our established threshold for success (a minimum cycle mean of ≥ 4/5), all cycles of ECHO-ONMH are considered successful in the appropriateness outcome.

### Cost

The estimated cost per person to participate in a 72-h conference is $5365, compared to $3581 to participate in the same number of CME hours for ECHO. The cost to travel to conferences might be paid by individuals, or they may have travel reimbursed through their work. The cost to participate in ECHO is almost entirely funded through the government, except for the cost of the participant’s technology peripherals, such as camera/microphones. Based on our cost comparison model, the cost savings for an individual to participate in a cycle of ECHO is about $25 per CME hour, $1784 per annual cycle, and $728,056 by model/programme per year. See Table 2 for summary of findings. While we have not determined a success threshold for this metric, as it is an exploratory measure, this does constitute a significant cost savings for both the individual and the public healthcare funder.

**Table 2:** Cost comparison between ECHO-ONMH and an in-person Toronto-based conference

Factors	Conference in Toronto	ECHO-ONMH	Notes for calculations
Total participants	408	408	408 total ECHO participants across four cycles (i.e. attended ≥ 1 session) Same number of participants used in order to compare two cases appropriately
Total registration cost per person	$2222.64	N/A	Average registration cost per CME conference hour per person = $30.87; $30.87/h × 72 h = $2222.64 for 72 h of conference CME No registration cost for ECHO-ONMH
Total average cost of travel per person	$442.81	N/A	Average cost estimated by taking total distance from participant organization site to and from Toronto, reimbursed at $0.41/Km
Total average cost for accommodations per person	$2700.00	N/A	$300/night × 9 nights = $2700 for hotel cost No hotel cost for ECHO-ONMH
Total cost of video camera per person	N/A	$53.13	Logitech video camera with built-in mic: $39.99 USD = $53.13 CAD (converted 23 June 2020) as per product page on Logitech website at time of writing No video camera needed for conference
Total operational/admin programme cost per person	N/A	$3528.00	Total programme cost per CME hour (as per programme funding agreement) = $360,429/72 h = $5005.95 Average of 102 people per session = $49 per CME hour $49/h × 72 total CME hours per person = $3528 Our assumption is that programme cost for conferences is built into the cost that the participant pays for registrations so it was not applicable in this line
Total cost for 72 CME hours per person	$5365.45	$3581.00	$1784 difference in cost per person, per year
Total cost per CME hour	$75.00	$50	$25 difference per CME hour
Total cost all participants	$2,189,104	$1,461,048	Total cost savings per 408-attendee CME learning programme = $728,056

### Feasibility

The average number of sessions participants attended within a given cycle was 7.34 (SD = 7.85, 32 sessions), 8.13 (SD = 7.27, 33 sessions), 12.53 (SD = 9.62, 33 sessions) and 15.78 (SD = 10.90, 36 sessions) for cycles 1, 2, 3 and 4 of ECHO-ONMH, respectively. When compared to the global average of six sessions from the ECHO Institute [4], all ECHO-ONMH cycles exceed the global average, with the numbers increasing incrementally by year, and can be considered successful for the feasibility outcome.

Further exploration of the feasibility outcome within ECHO showed that the proportion of participants that attended the recommended ≥ 60% sessions ranged from 10 to 36% across the four cycles. Full breakdown by cycles is reported in Appendix A. The two professional groups that most often attended ≥ 60% sessions were social workers/counsellors/case managers (46%), followed by nurse practitioners (35%). Most of the participants that attended ≥ 60% sessions worked in family health teams (27%), hospitals (19%), community health centres (19%), community mental health and addiction centres (15%) and nurse practitioner-led clinics (10%). Most of the participants that attended ≥ 60% sessions worked in organizations located geographically in the Champlain (19%), North East (18%), North West (16%) and Erie St Clair (12%) LHINs, which notably have fairly rural and/or underserved populations.

ECHO-ONMH encourages interprofessional learning with participants sharing perspectives from diverse practice settings and locations of practice. For those participants that attended ≥ 60% sessions each cycle, diversity with regard to profession (7 different professions), practice type (9 different practice types) and regional areas of practice (12 different LHINs) was still present. The full distribution of professions, practice types and regions for participants that attended ≥ 60% sessions is located in Appendix.

### Fidelity

We evaluated the recordings of five randomly selected sessions per cycle (20 total) for adherence to the four ECHO principles and the presence of the “all teach, all learn” environment, and found that all assessed sessions for each cycle met the fidelity criteria (5/5 or 100%). Given our fidelity threshold (100% of criteria are met), all four ECHO-ONMH cycles had exceptionally high rates of fidelity to the Project ECHO model.

### Penetration

**Fig. 2:**
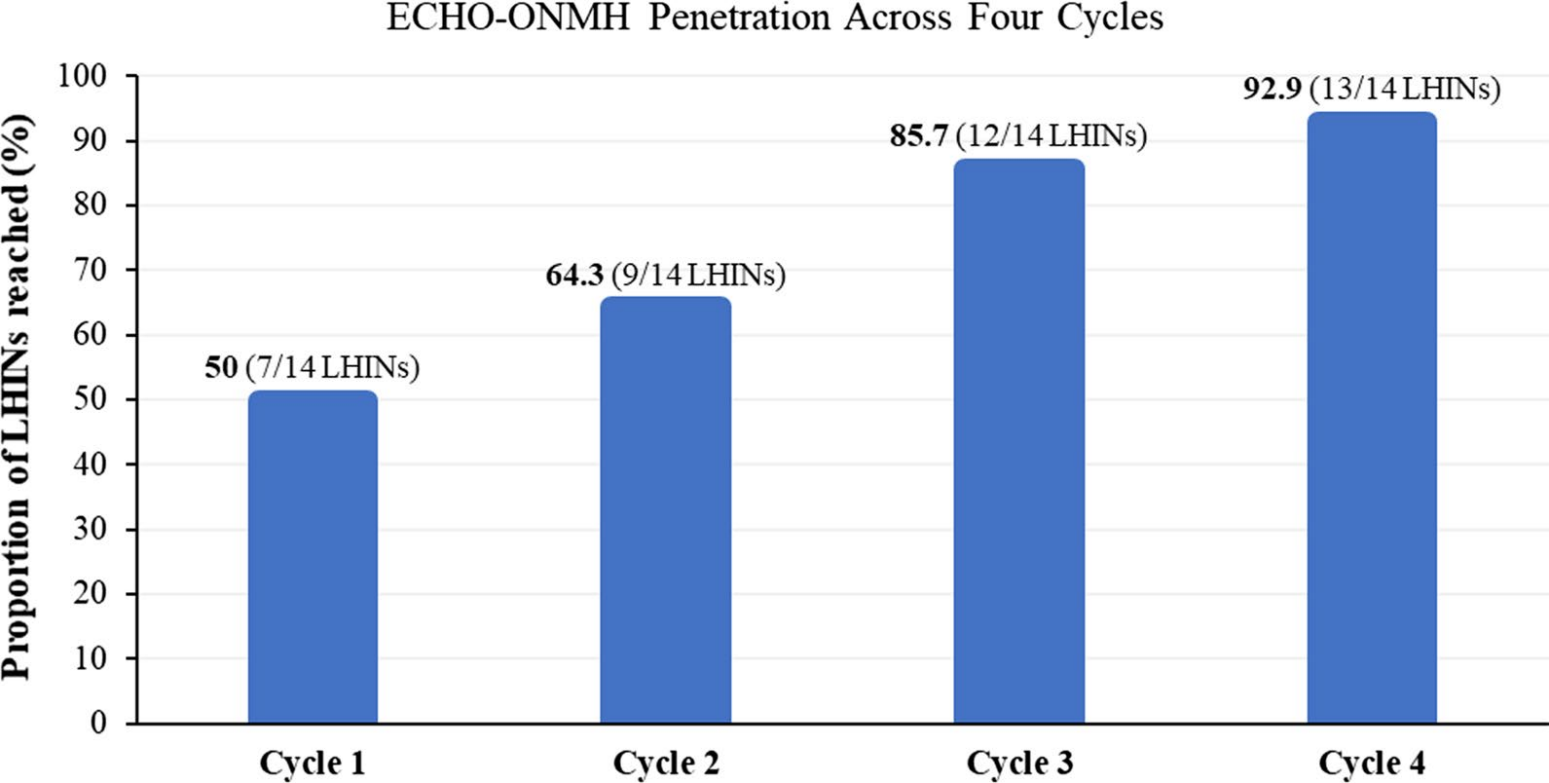
Penetration across four cycles of ECHO-ONMH

Penetration (proportion of the 14 LHINs reached) across each cycle can be seen in Fig. 2. For cycle 1, 50% (7/14 LHINs) penetration was observed, with a range of 8–67 PCP registrants across LHINs. For cycle 2, 64.3% (9/14 LHINs) penetration was observed, with a range of 2–61 PCP registrants across LHINs. For cycle 3, 85.7% (12/14 LHINs) penetration was observed, with a range of 1–21 PCP registrants across LHINs. For cycle 4, 92.9% (13/14 LHINs) penetration was observed, with a range of 1–23 PCP registrants across LHINs. When comparing these proportions to our established success threshold for penetration (i.e. 100% LHINs have been reached each cycle), none of the four cycles of ECHO-ONMH achieved successful penetration; however, an ongoing increase in LHIN penetration each cycle was observed, with cycles 3 and 4 being close to reaching successful penetration.

### Sustainability

All four ECHO-ONMH cycles achieved and surpassed minimum adoption. As described in the findings for adoption, each cycle had more than 25 PCPs registered and a roster of more than 20 participants, and exceeded the minimum average of six participants per session. By surpassing adoption thresholds year over year (cycles 1 to 4), the programme met all funder agreement requirements, and as such sustained funding to support the model. Therefore, ECHO-ONMH appears to be a sustainable programme.

## Discussion

Our study describes how Proctor et al.’s eight implementation outcomes were adapted to assess the implementation of an ECHO project, specifically ECHO-ONMH, a virtual capacity-building programme implemented in Ontario, Canada. We identified specific implementation outcome measures for ECHO-ONMH and used these measures to evaluate its implementation success from 2015 to 2019, showing that the ECHO model at CAMH was implemented successfully.

Implementation success thresholds for acceptability and appropriateness were surpassed for all 4 years, showing strong signs of sustainability. Although the success threshold for adoption was exceeded for all four cycles, a decrease in number of people registered for ECHO-ONMH was observed over the years. We believe this is largely because registration requirements changed, where individuals were required to register, instead of clinics registering them on their behalf. An additional possibility is that as more and more people participate in ECHO, a saturation point will occur and participation will continue to decline; however, at the time of this study, adoption for ECHO-ONMH remains high. Also of note, while the number of registrants decreased, the conversion rate of those who registered compared to those who attended one or more sessions increased each year. Feasibility scores surpassed the predetermined success thresholds, and increased incrementally each year; by the fourth cycle, participants were joining ECHO-ONMH for an average of 16 sessions. The professions that were more likely to join an ECHO session were social workers or nurse practitioners, both typically salary-based healthcare professions in Ontario and most frequently part of a team. Based on the feasibility data, it seems likely that the ECHO-ONMH model is most feasible for salary-based providers, because they are not required to forgo income in order to join the sessions, and they are also part of team-based organization that can help share responsibility for patients, enabling participants to join the sessions. Compared to in-person conferences, ECHO-ONMH was found to be more cost-effective, at a cost of $50 per hour of CME compared to $75 per hour of CME for in-person training at conferences. All randomly selected sessions showed complete fidelity to the model by meeting all five criteria as set out by the ECHO Institute each cycle. Of note, penetration was the only outcome in which the ECHO-ONMH programme did not meet the predetermined success thresholds (i.e. reaching all 14 regions per cycle). Additional outreach ensuring that the programme is advertised and reaches PCPs across the province will support a diverse set of participants, and will also ensure that all regions have access to mental health support within primary care. While ECHO-ONMH was successful in most of these outcomes, we believe that this framework is important for new and developing ECHOs, especially those that are experiencing implementation challenges such as low rates of adoption or fidelity, to identify areas for improvement early on in the programme implementation. This framework can help them pinpoint issues and support the identification of quality improvement initiatives early on in the implementation process.

While numerous studies have looked at patient and provider outcomes for the ECHO model, few have analysed whether the ECHO model was successfully implemented as designed in their setting [10, 17]. This study provides a measurement framework for other ECHO programmes, as well as other capacity-building or educational models (virtual or not), to evaluate implementation outcomes when replicating interventions. Understanding whether the model has been implemented as expected can ensure that the associated patient and/or provider outcomes that are being observed are a result of the planned intervention. Additionally, this measurement framework can help support ongoing quality improvement efforts by providing baseline implementation outcomes.

While a few non-ECHO studies have used similar approaches to those articulated in this study to describe elements such as feasibility, acceptability, appropriateness, penetration and cost, the research on these outcomes for virtual care remains a relatively new, albeit fast-growing, body of literature [10, 25, 27–36]. Some ECHO and virtual capacity-building projects do report on one or two aspects of implementation, such as satisfaction or participation, but do not provide an overall implementation outcome framework (including benchmarks) rooted in implementation science [10, 17, 24]. To our knowledge, this study is the first to describe all eight outcomes of implementation for an ECHO or a similar virtual capacity-building programme. While the implementation outcome measures and thresholds used in this study are specific to ECHO-ONMH and would have to be adapted for each ECHO context, the adapted ECHO implementation outcomes (see column 2 in Table 1) are largely applicable to most ECHOs. Further, the findings in this study provide a foundation for ECHO interventions, as well as other educational and administrative interventions, to gauge how they conceptualize success with respect to implementation.

The applicability of this study extends beyond the ECHO context. With the recent disruption caused by the COVID-19 pandemic, and the need to exchange information both rapidly and remotely, the ability to effectively implement virtual models of care and capacity-building is essential [37, 38]. Having a framework with concrete measures to gauge how well an intervention is implemented can provide clarity and context when exploring intervention effectiveness, support quality improvement, and lend support for the causality of outcomes to the intervention.

There are several limitations to consider when interpreting our findings. First, the data and measures used for this study were obtained from a single ECHO programme with a specific focus area for one province in Canada; as such, generalizability of our specific findings to other settings remains unclear. Future research will aim to validate this framework with additional ECHO projects, and other diverse virtual interventions. In addition, implementation success may be defined differently for each ECHO programme; therefore, success thresholds will be determined based on planned implementation outcomes for each project. Additionally, the cost outcome was measured at a very high level, but additional robust economic analysis on the ECHO model is warranted. An additional limitation is that the programme did not have this framework established prior to implementation, so measures were not created specifically to assess implementation outcomes. While this study was able to identify numerous measures that aptly measured implementation outcomes, a prospective approach rather than a retrospective approach would likely be beneficial. As such, this framework will be used to help guide reporting and surveys for future ECHOs. Lastly, survey data, such as the data used for the appropriate and acceptability measures, only represent the individuals who attended the sessions and opted to complete the weekly session satisfaction surveys. This may introduce a response bias, whereby those who completed the satisfaction surveys may have been more likely to respond with higher scores, resulting in higher mean ratings for cycles. Additionally, the response rates for these surveys were low; however, this is common with survey collection, especially online surveys, were average response rates are around 29% [39, 40]. The ECHO-ONMH team recognized this challenge and had utilized various means to encourage weekly completion of the survey, such as acknowledging participant feedback during sessions as it informs improvement initiatives, and providing continuing medical education credits to participants that complete the survey.

Also of note, the authors believe that both in-person educational models and ECHO are important and that a blend of the two is important to learners, and while this estimate considers strictly direct costs, a more robust economic analysis that includes direct/indirect time, a sensitivity analysis and a cost–benefit or cost-consequence analysis would provide a more accurate analysis of the true cost comparison.

## Conclusions

In summary, this study described implementation success for a specific virtual mental health-focused capacity-building model. Based on measures established for each of Proctor et al.’s eight implementation outcomes [16], the ECHO-ONMH programme has shown high levels of implementation success in all areas but penetration, where the programme has some additional work to do with regard to targeted recruitment with the particular region in Ontario. The implementation measures described in this study provide a means for educational or administrative interventions to evaluate their success with respect to implementation outcomes. The findings from this study act as a benchmark for other ECHOs to compare their success through an implementation lens, and can provide an opportunity for programmes to prospectively establish measures and benchmarks that will help assess implementation success. Additional research should build on these initial findings to understand the validity and reliability of these ECHO-identified implementation outcome measures and success thresholds for diverse ECHO programmes, and other virtual education or capacity-building models. A future study will partner with diverse international ECHOs to validate the adapted implementation outcomes/measures/thresholds and collectively establish additional general ECHO measures and thresholds.

## Data Availability

The data sets generated and/or analysed during the current study are not publicly available due to programmatic confidentiality and privacy agreements in place.

## Appendix

See Table 3.

**Table 3:** Distribution of professions, practice types and regions for participants that attended ≥ 60% sessions across four cycles of ECHO-ONMH

	Cycle 1 (*N* = 146)	Cycle 2 (*N* = 113)	Cycle 3 (*N* = 58)	Cycle 4 (*N* = 91)	Total (*N* = 408)
Proportion of participants who attended ≥ 60% sessions	20 (14%)	11 (10%)	15 (26%)	33 (36%)	79 (19%)

Distribution of professions that attended ≥ 60% sessions	Cycle 1 (*N* = 20)	Cycle 2 (*N* = 11)	Cycle 3 (*N* = 15)	Cycle 4 (*N* = 33)	Total (*N* = 79)
Social worker/counsellor/case manager	13 (65%)	5 (45%)	4 (27%)	14 (42%)	36 (46%)
Nurse practitioner	6 (30%)	4 (36%)	5 (33%)	13 (39%)	28 (35%)
Physician	0	1 (9%)	4 (27%)	2 (6%)	7 (9%)
Pharmacist	1 (5%)	0	1 (7%)	0	2 (3%)
Registered dietician	0	0	1 (7%)	0	1 (1%)
Registered nurse	0	1 (9%)	0	3 (9%)	4 (5%)
Psychotherapist	0	0	0	1 (3%)	1 (1%)

Distribution of participants by practice type that attended ≥ 60% sessions	Cycle 1 (*N* = 20)	Cycle 2 (*N* = 11)	Cycle 3 (*N* = 15)	Cycle 4 (*N* = 33)	Total (*N* = 79)
Family health team/family health centre/family practice	7 (35%)	5 (45%)	7 (47%)	2 (6%)	21 (27%)
Community health centre	3 (15%)	2 (18%)	0	10 (30%)	15 (19%)
Nurse practitioner-led clinic	4 (20%)	1 (9%)	1 (7%)	2 (6%)	8 (10%)
Community mental health and addiction services	6 (30%)	0	2 (13%)	4 (12%)	12 (15%)
Private practice/solo practitioner	0	1 (9%)	1 (7%)	0	2 (3%)
University mental health centre/wellness centre	0	1 (9%)	0	1 (3%)	2 (3%)
Health Canada-First Nations & Inuit Health Branch Ontario	0	0	2 (13%)	0	2 (3%)
Hospital	0	0	1 (7%)	14 (42%)	15 (19%)
Community support services	0	0	1 (7%)	0	1 (1%)
Aboriginal Health Access Centre		1 (9%)			1 (1%)

Distribution of participants by LHIN that attended ≥ 60% sessions	Cycle 1 (*N* = 20)	Cycle 2 (*N* = 11)	Cycle 3 (*N* = 15)	Cycle 4 (*N* = 33)	Total (*N* = 79)
Central West	4 (20%)	1 (9%)	0	2 (6%)	7 (9%)
North East	8 (40%)	2 (18%)	2 (13%)	2 (6%)	14 (18%)
North West	7 (35%)	4 (36%)	0	2 (6%)	13 (16%)
Erie St Clair	1 (5%)	0	5 (33%)	3 (9%)	9 (12%)
Hamilton Niagara Haldimand Brant	0	2 (18%)	0	3 (9%)	5 (6%)
North Simcoe Muskoka	0	2 (18%)	0	3 (9%)	5 (6%)
South West	0	0	0	3 (9%)	3 (4%)
Waterloo Wellington	0	0	1 (7%)	0	1 (1%)
Champlain	0	0	5 (33%)	10 (30%)	15 (19%)
Central East	0	0	1 (7%)	2 (6%)	3 (4%)
Central	0	0	1 (7%)	0	1 (1%)
Toronto Central	0	0	0	3 (9%)	3 (4%)

## Abbreviations

ECHO: Extension for Community Healthcare Outcomes
PCP: Primary care providers
ECHO-ONMH: ECHO Ontario Mental Health
CAMH: Centre for Addiction and Mental Health
SoC: Statement of collaboration
CME: Continuing medical education
LHIN: Local health integration network
CAD: Canadian dollar

## Footnotes

1 For cycle 2, the way in which participation was recorded was slightly different from that in cycle 1. In order to ensure consistency in the way in which the data were recorded, the authors used sourced data to validate the participant attendance log. The authors reviewed saved Zoom video recordings and chat threads for each session, which included participant introductions. Attendance surveys that were used to capture participant attendance via the online data capture platform REDCap were also reviewed to validate the attendance log for cycle 2.
